# Supplementary material for: Pemafibrate Ameliorates Steatotic Liver Disease Regardless of Endothelial Dysfunction in Mice
Source: Antioxidants (Basel). 2025 Jul 20;14(7):891. doi: 10.3390/antiox14070891 (PMC12292507; doi:10.3390/antiox14070891)
Supplement: Supplementary file 1 [file antioxidants-14-00891-s001.zip › antioxidants-3716762-supplementary.pdf]

Table S1. Primers used in this study

Gene Symbol (Synonyms)	Description	Forward	Reverse	NCBI reference sequence
Itgax (CD11c)	integrin alpha X	ATTTCTGAGAGCCCAGACGA	CCATTTGCTTCCTCCAACAT	NM_021334.3
Adgre1 (F4/80)	adhesion G protein-coupled receptor E1	TGCATCTAGCAATGGACAGC	GCCTTCTGGATCCATTTGAA	NM_001355722.2
Ccl2 (Mcp-1)	C-C motif chemokine ligand 2	CCAACTCTCACTGAAGCCAGCTC	TTGGGATCATCTTGCTGGTGAA	NM_011333.3
Nos2 (iNOS)	inducible nitric oxide synthase	CACCAAGCTGAACTTGAGCG	CGTGGCTTTGGGCTCCTC	NM_001313921.1
Cd163	Cluster of Differentiation 163 antigen	TGGTGTGCAGGGAATTACAA	ATCCCTGCTGTGGGTACAAG	NM_001170395.1
Mrc1 (Cd206)	mannose receptor, C type 1	CAAGGAAGGTTGGCATTTGT	CCTTTCAGTCCTTTGCAAGC	NM_008625.2
Cd209a	Cluster of Differentiation 209a antigen	TGCTGGTTGTCATCCTTGTC	GATCTACGCCAGCCTTCAAC	NM_133238.5
Cxcl1	C-X-C motif chemokine ligand 1	ACTGCACCCAAACCGAAGTC	TGGGGACACCTTTTAGCATCTT	NM_008176.3
Cxcl2	C-X-C motif chemokine ligand 2	GCGCCCAGACAGAAGTCATAG	GGCAAACTTTTTGACCGCC	NM_009140.2
Clec4e (mincle)	C-type lectin domain family 4 member E	ACCAAATCGCCTGCATCCCA	GTGGCTGTAAGTTCTGCCCG	NM_019948.2
Ccn2 (Ctgf)	cellular communication network factor 2	ACCCGAGTTACCAATGACAATACC	CCGCAGAACTTAGCCCTGTATG	NM_010217.2
Tgfb1	transforming growth factor- beta 1	GTGTGGAGCAACATGTGGAACTCTA	TTGGTTCAGCCACTGCCGTA	NM_011577.2
Col1a1	collagen, type I, alpha 1	ACTCAGCCGTCTGTGCCTCA	GGAGGCCTCGGTGGACATTA	NM_007742.4
Col3a1	collagen, type III, alpha 1	TGGTTTCTTCTCACCCTTCTTC	GTCCATGGCCATCAGGAAGC	NM_009930.2
Rac1	Rac family small GTPase 1	ATGTGACTAATGCTGCCTCTAGACC	ACAGCGTTAGCACTAGCAGTTGAC	NM_001347530.1
Cyba (p22 phox)	cytochrome b-245 alpha chain	TGGCTACTGCTGGACGTTTCAC	CTCCAGCAGACAGATGAGCACAC	NM_007806.3
Cybb (gp91 phox)	cytochrome b-245 beta chain	TTGAAACCACACCTAAGCCATCTG	AACTGAGGCTTGAGACAACCTGGTA	NM_007807.5
Ncf1 (p47 phox)	neutrophil cytosolic factor 1	GTGCCCAAAGATGGCAAGAATAAC	GCAATGGCCCGATAGGTCTG	NM_001286037.1
Ncf2 (p67 phox)	neutrophil cytosolic factor 2	AAGCAGAAGCTGTTCGAGCC	CGGCACAAAGCCAAACAATA	NM_010877.6
Ncf4 (p40 phox)	neutrophil cytosolic factor 4	TCAGCCAACATCGCTGACA	TTGACCTCGATGACAAAAACAAA	NM_008677.3
Nox1	NADPH oxidase 1	CATCCAGTCTCCAAACATGACA	GCTACAGTGGCAATCACTCCAG	NM_172203.2
Nox4	NADPH oxidase 4	TGCTCATTTGGCTGTCCCTA	TGCAGTTGAGGTTCAGGACA	NM_015760.5
Noxo1	NADPH oxidase organizer 1	TGAGTGCCATCCAGAGTCGAT	AACCCGAGTCCCTTGTTCCT	NM_027988.4
Il1b	interleukin 1 beta	ATGGCAACTGTTCCTGAA	GTACAAAGCTCATGGAGA	NM_008361.4
Il6	interleukin 6	TTCCCTACTTCACAAGTC	GGTTTGCCGAGTAGATCT	NM_031168.2
Tnf	tumor necrosis factor	GTTCTATGGCCCAGACCCTCAC	GGCACCACTAGTTGGTTGTCTTTG	NM_013693.3
Infγ	interferon gamma	ATGAACGCTACACACTGCATC	CCATCCTTTTGCCAGTTCCTC	NC_000076.7
Nfe2l2 (Nrf2)	NF-E2-related factor 2	TCACACGAGATGAGCTTAGGGCAA	TACAGTTCTGGGCGGCGACTTTAT	NM_010902.5
Keap1	kelch-like ECH associated protein 1	AGCAGATCGGCTGCACTGAA	AGCTGGCAGTGTGACAGGTTG	NM_001110305.1
Hmox1 (HO1)	heme oxygenase 1	TAAGACCGCCTTCCTGCTCAACAT	TGCTGGTTTCAAAGTTCAGGCCAC	NM_010442.2
Pparα	peroxisome proliferator-activated receptor alpha	CTCAGGGTACCACTACGGAGTTCAC	TGAATCTTGCAGCTCCGATCAC	NM_001113418.1
Igf1	insulin-like growth factor 1	TGAGCTGGTGGATGCTCTTCAGTT	TCATCCACAATGCCTGTCTGAGGT	NM_001111274.1
Acox1	acyl-Coenzyme A oxidase 1, palmitoyl	CTGAGTAGCTGGCTTTGTGGTAA	AGAATGATTGTAGAGGCTGCTCTG	NM_015729.4
Srebf1 (SREBP1)	sterol regulatory element binding transcription factor 1	ACTGTCTTGGTTGTTGATGAGCTGGAGCAT	ATCGGCGCGGAAGCTGTCGGGGTAGCGTC	NC_000077.7
Foxo1	forkhead box O1	CTTCAAGGATAAGGGCGACA	GACAGATTGTGGCGAATTGA	NM_019739.3
Foxo3	forkhead box O3	GCTAAGCAGGCCTCATCTCA	TTCCGTCAGTTTGAGGGTCT	NM_001376967.1
Foxo4	forkhead box O4	AAGGACAAGGGTGACAGCAA	CTGTGCAAGGACAGGTTGTG	NM_018789.2
Flt1	FMS-like tyrosine kinase 1	ACACCGCGGTCTTGCCTTAC	AGTCTGGCCTGCTTGCATGA	NM_001363135.1
Gapdh	glyceraldehyde-3-phosphate dehydrogenase	TGTGTCCGTCGTGGATCTGA	TTGCTGTTGAAGTCGCAGGAG	NM_008084.4

Figure S1

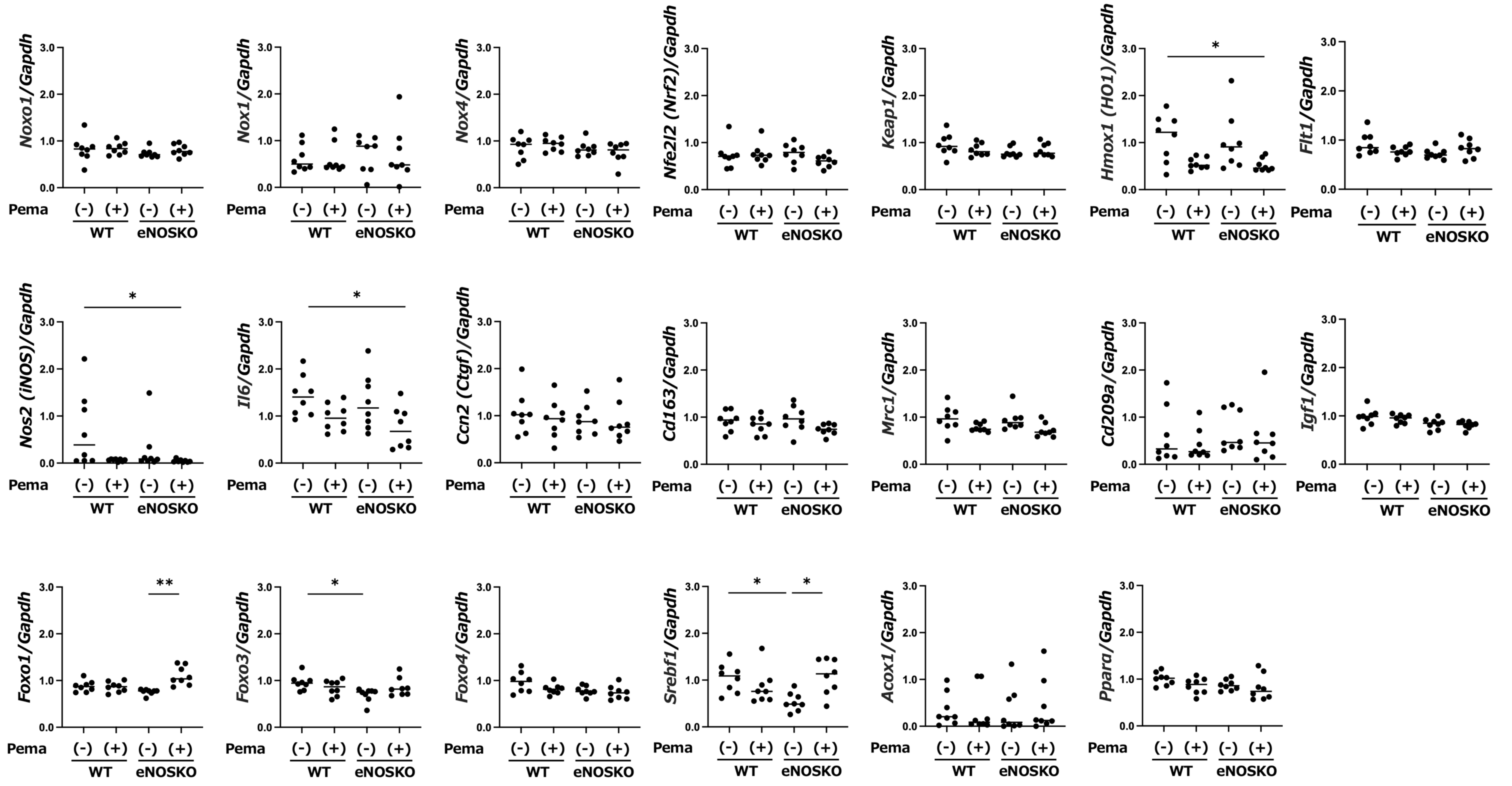

Noxo1/Gapdh
Nox1/Gapdh
Nox4/Gapdh
Nfe2l2 (Nrf2)/Gapdh
Keap1/Gapdh
Hmox1 (HO1)/Gapdh
Flt1/Gapdh
Nos2 (iNOS)/Gapdh
Il6/Gapdh
Ccn2 (Ctgf)/Gapdh
Cd163/Gapdh
Mrc1/Gapdh
Cd209a/Gapdh
Igf1/Gapdh
Foxo1/Gapdh
Foxo3/Gapdh
Foxo4/Gapdh
Srebf1/Gapdh
Acox1/Gapdh
Ppara/Gapdh
3.0
2.0
1.0
0.0
Pema
(-)
(+)
WT
eNOSKO
*
**

Figure S2

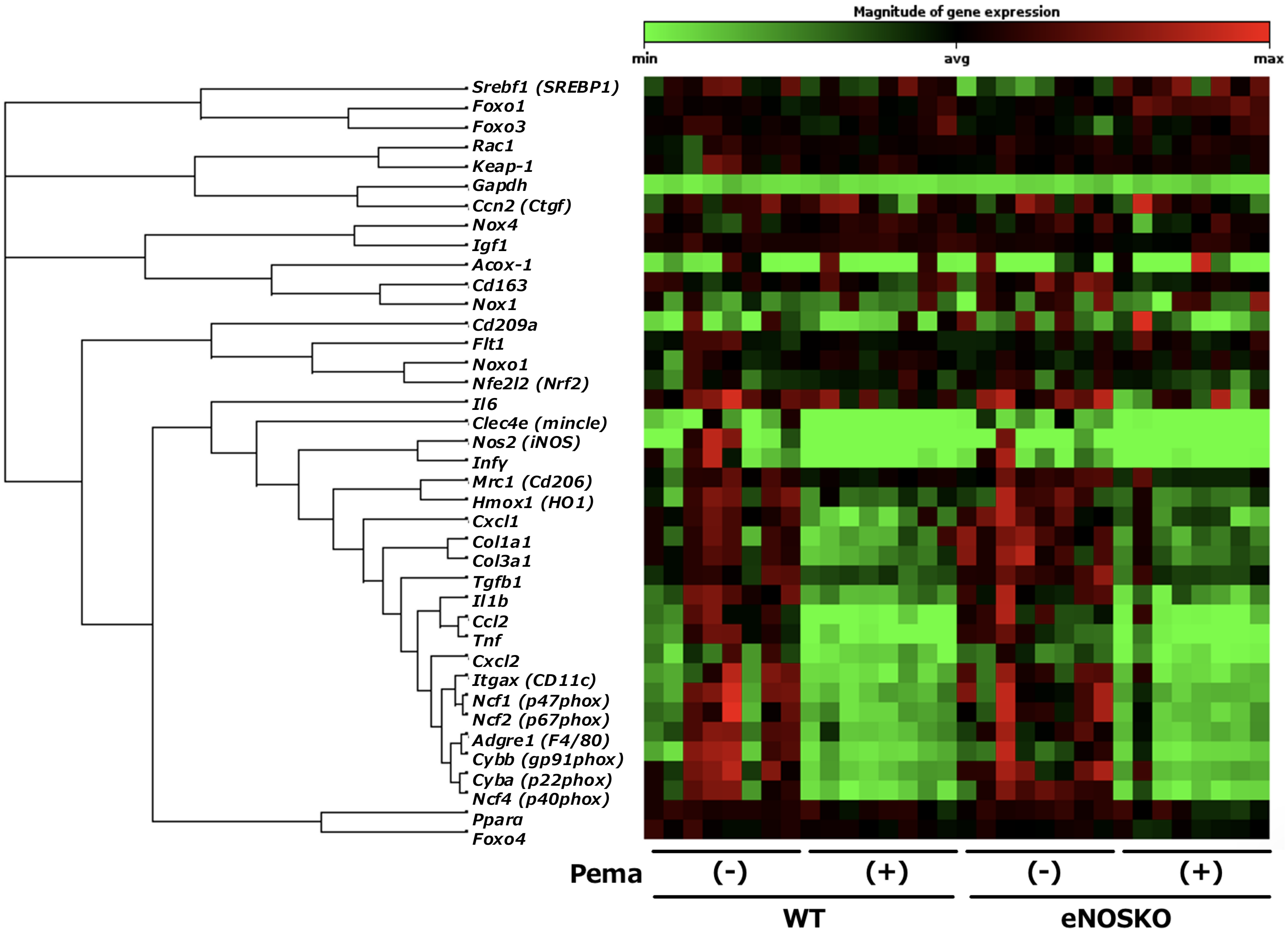

MASLD-associated candidate gene expression clustergram in vehicle-treated MASLD mice and pemafibrate-treated MASLD mice with and without eNOS deficiency., n = 8 mice per experimental group

Figure S3

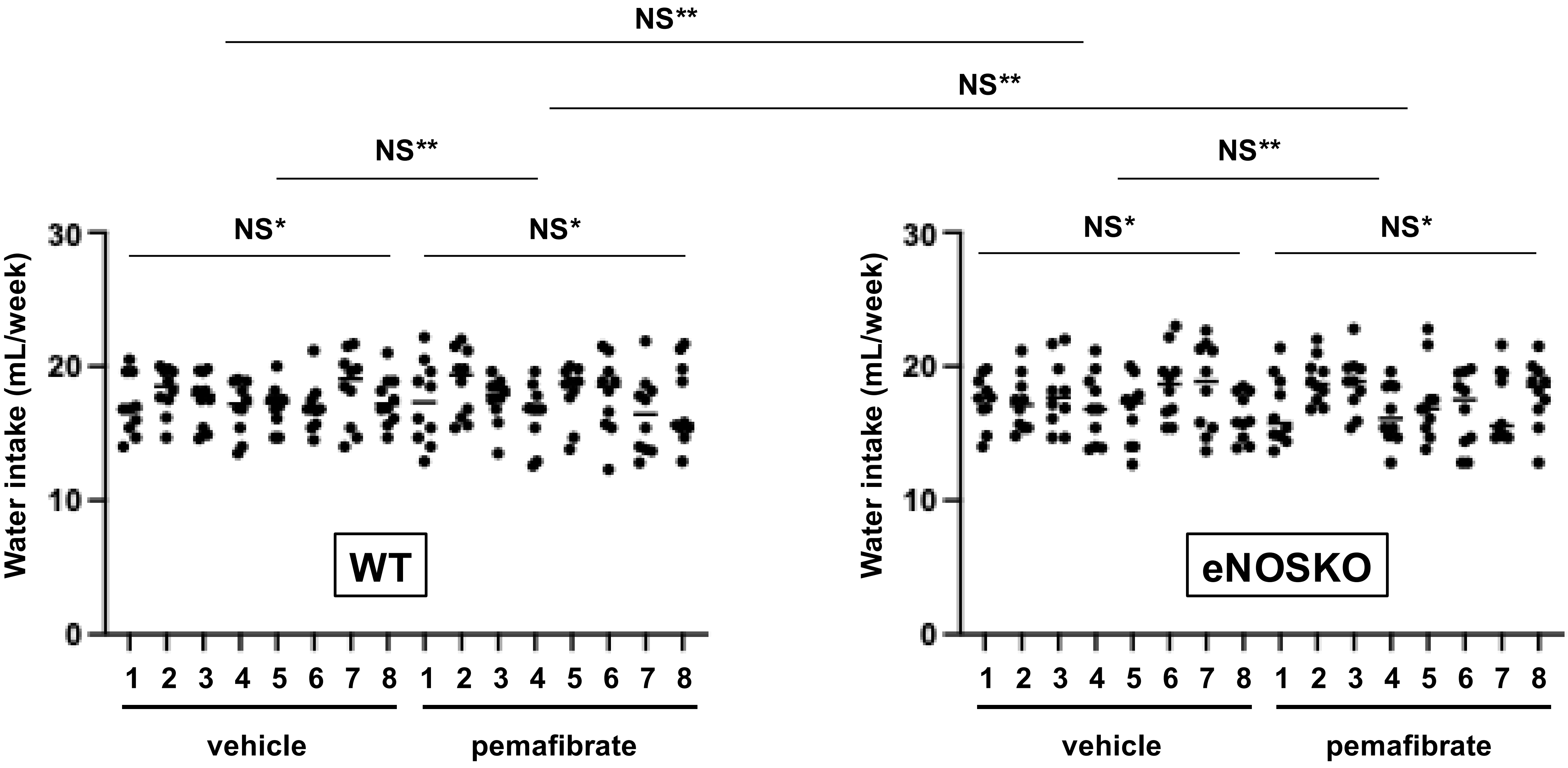

Average 17.4±2.0 mL/week 17.4±2.4 mL/week

Average 17.4±2.3 mL/week 17.5±2.4 mL/week

Weeky water intake during 10 weeks in each mouse

*Kruskal-Wallis test
**Mann-Whitney U test

Figure S4

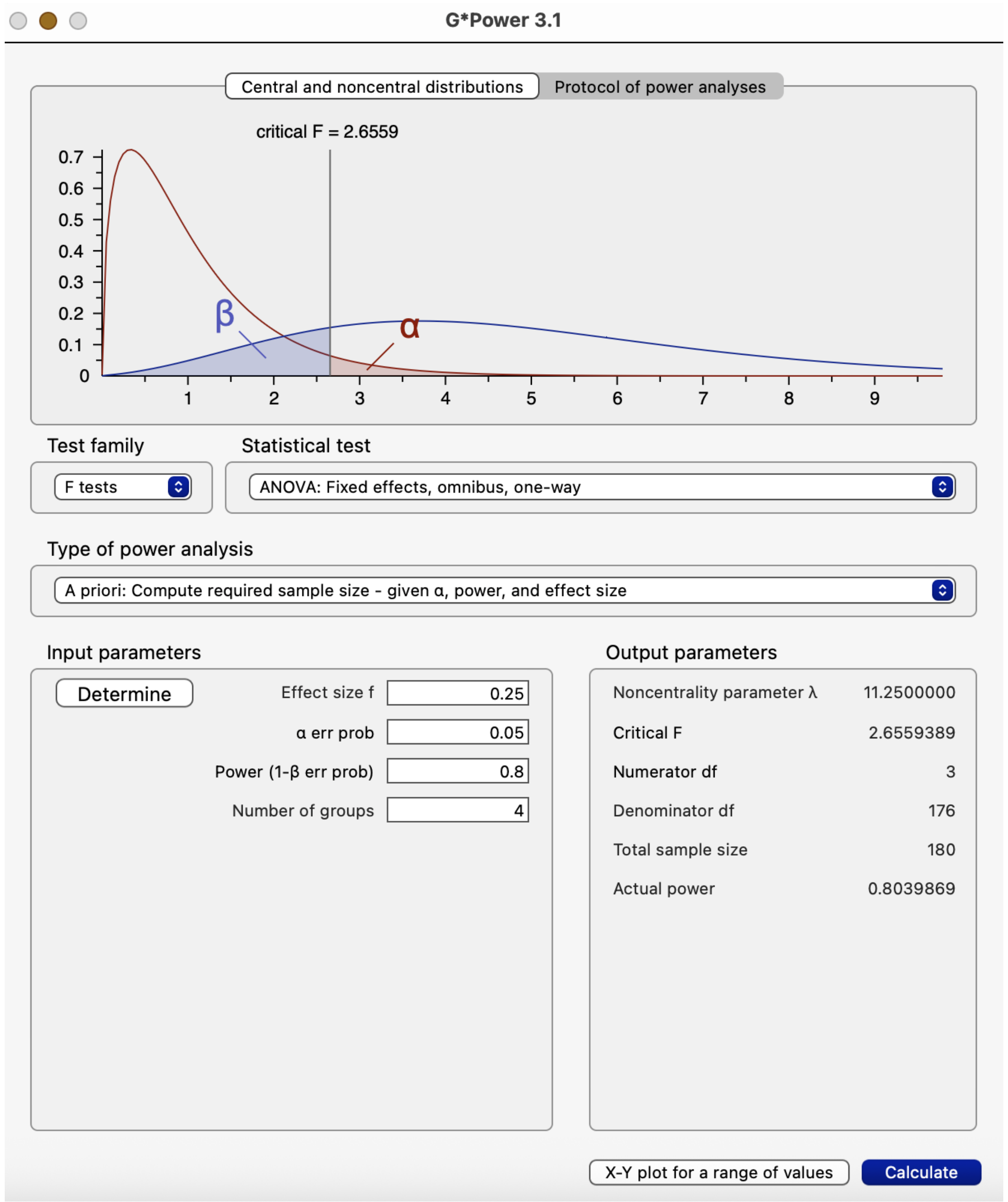

G*Power 3.1
Central and noncentral distributions
Protocol of power analyses
critical F = 2.6559
0.7
0.6
0.5
0.4
0.3
0.2
0.1
0
1
2
3
4
5
6
7
8
9
β
α
Test family
F tests
Statistical test
ANOVA: Fixed effects, omnibus, one-way
Type of power analysis
A priori: Compute required sample size - given α, power, and effect size
Input parameters
Determine
Effect size f 0.25
α err prob 0.05
Power (1-β err prob) 0.8
Number of groups 4
Output parameters
Noncentrality parameter λ 11.2500000
Critical F 2.6559389
Numerator df 3
Denominator df 176
Total sample size 180
Actual power 0.8039869
X-Y plot for a range of values
Calculate
